# Detection of Potential Drug-Drug Interactions for Outpatients across Hospitals

**DOI:** 10.3390/ijerph110201369

**Published:** 2014-01-27

**Authors:** Yu-Ting Yeh, Min-Hui Hsu, Chien-Yuan Chen, Yu-Sheng Lo, Chien-Tsai Liu

**Affiliations:** 1 Graduate Institute of Medical Sciences, College of Medicine, Taipei Medical University, 250 Wuhsing St., Taipei 110, Taiwan; E-Mail: ting@shh.org.tw; 2 Information Technology Office, Shuang Ho Hospital, Taipei Medical University, 291 Zhongzheng Rd., New Taipei City 235, Taiwan; 3 Graduate Institute of Biomedical Informatics, College of Medical Science and Technology, Taipei Medical University, 250 Wuhsing St., Taipei 110, Taiwan; E-Mails: ccnsdoctor@mohw.gov.tw (M.-H.H.); loyusen@gmail.com (Y.-S.L.); 4 Department of Information Management, Wan Fang Hospital, Taipei Medical University, Section 3, 111 HsingLong Rd., Taipei 116, Taiwan; E-Mail: cychen12@vghtpe.gov.tw

**Keywords:** drug-drug interactions, health smart cards, CPOE system, system interoperability, patient safety

## Abstract

The National Health Insurance Administration (NHIA) has adopted smart cards (or NHI-IC cards) as health cards to carry patients’ medication histories across hospitals in Taiwan. The aims of this study are to enhance a computerized physician order entry system to support drug-drug interaction (DDI) checking based on a patient’s medication history stored in his/her NHI-IC card. For performance evaluation, we developed a transaction tracking log to keep track of every operation on NHI-IC cards. Based on analysis of the transaction tracking log from 1 August to 31 October 2007, physicians read patients’ NHI-IC cards in 71.01% (8,246) of patient visits; 33.02% (2,723) of the card reads showed at least one medicine currently being taken by the patient, 82.94% of which were prescribed during the last visit. Among 10,036 issued prescriptions, seven prescriptions (0.09%) contained at least one drug item that might interact with the currently-taken medicines stored in NHI-IC cards and triggered pop-up alerts. This study showed that the capacity of an NHI-IC card is adequate to support DDI checking across hospitals. Thus, the enhanced computerized physician order entry (CPOE) system can support better DDI checking when physicians are making prescriptions and provide safer medication care, particularly for patients who receive medication care from different hospitals.

## 1. Introduction

It is common for patients who visit multiple hospitals with the same or similar conditions to change doctors (or hospitals) in Eastern cultures [1,2,3,4]. The prevalence of patients who visited multiple hospitals with the same or similar condition was nearly 40% among patients attending government outpatient departments in Hong Kong [1], 23% among primary care patients in Japan [2,3], and 23.5% among outpatients in Taiwan [4,5,6]. Previous studies have found that patients who receive medical care from multiple health care providers, particularly from different hospitals, are more likely to suffer adverse drug reactions (ADRs) [7,8,9,10]. Repeats sentence above ADRs, which are harmful, unintended reactions to medicines that occur at doses normally used for treatment, are a major cause of morbidity and mortality and lead to increased hospitalizations and medical costs [11,12,13]. Repeats sentence above About 25% of ADRs can be attributed to DDIs [14,15]. Thus, DDIs remain an important issue in clinical practice and drug management. Repeats sentence above

Although it is impossible to recognize every potential DDI when prescribing or dispensing, in most cases DDIs are predictable and preventable by using well-established, currently available DDI databases [12,16,17]. There have been various approaches proposed to reduce the risk of ADRs, including DDIs, using information communication technologies (ICTs). Some researchers suggested that pharmacists use computerized screening software to identify potential drug therapy problems and prevent adverse events [15,18,19]. Others suggested that physicians use computerized provider order entry (CPOE) with clinical decision support (CDS) to improve medication errors [20,21,22,23,24,25]. Evidence has shown these approaches can identify potential ADRs (including DDIs) and improve medication safety. However, the approaches were unlikely to be effective in detection of potential DDIs for the patients who frequently visit multiple hospitals because of lack of a complete medication history across hospitals.

In Taiwan, the National Health Insurance Administration (NHIA) has adopted smart cards (or NHI-IC cards) as health cards since November 1999 [26,27,28]. Patients with their NHI-IC cards can freely access nationwide hospitals or clinics contracted with the BNHI for medical services. The rate of contracted healthcare providers is over 99% of total healthcare providers in Taiwan. Up to 60 entries of the most up-to-date prescribed medicines are stored in a patient’s NHI-IC card that is as vehicle of personal health insurance information. The health care provider is responsible for writing the prescriptions into the card to disclose patient medical information appropriately. It was used as a reference when doctors treat a patient so that they don’t duplicate prescriptions. This helps safeguard patient safety, improve health care quality and facilitate interaction of physician-patient relationship [29]. Therefore, insured patients can own themselves medication record with NHI-IC cards. In regard to informed consent and autonomy, patients have rights to release their medical information or not. Under the direct user's consent and autonomy, patient can present his/her NHI-IC card to a health care provider when receiving a medical service. Since there are around 23 million NHI-IC cards currently in use across Taiwan, we used the NHI-IC cards as a common infrastructure for sharing patients’ medication history across hospitals, and enhanced CPOE systems to access the NHI-IC cards. Based on the medication history, the enhanced CPOE system can support DDI checking across hospitals when physicians prescribe medicines for patients. In our previous work we had enhanced CPOE systems to detect potential duplicate medications for the patients who receive medication care from multiple hospitals [30]. In this work, we focused on addressing the DDI problems involving multiple hospitals.

## 2. System Framework for Enhancing DDI Checking Involving Multiple Hospitals

**Figure 1:**
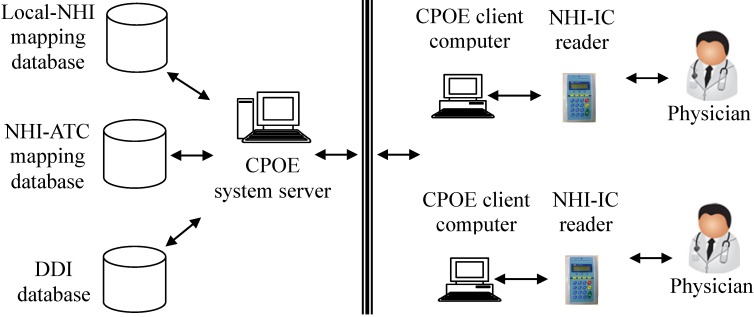
The framework of potential DDIs detection across hospitals.

The system framework is shown in Figure 1. A CPOE system usually consists of several client computers and a server. The client directly interacts with physicians for prescriptions, orders and results management. The server handles client requests and performs database transactions. The client was enhanced with the capability to read/write NHI-IC cards. Up to 60 prescribed drugs can be stored in the NHI-IC card [26,28]. Each prescribed drug item consists of prescribed date, drug code (represented by an NHI code), number of days (to be taken), dosage and frequency of use. It is a health care provider’s responsibility to write prescribed drugs into a patient’s NHI-IC card. Thus, a physician with the enhanced CPOE system can access a patient’s medication history stored in his/her NHI-IC card no matter where the patient receives medical care in Taiwan. Since it took about 20 s to read patient records from a NHI-IC card, and the average time in writing prescriptions into NHI-IC card is 1 min and 53 s, we decided to improve the workflow of DDI checking. We make changes to the procedure of writing NHI-IC cards. Initially the card writing procedure was performed by physicians after their prescriptions were completed. After the change, the procedure was performed by cashiers at checkout counters. This change reduced the physicians’ workload and service time.

Both security and privacy of electronic medical records are significant priority issues [31]. The NHI-IC card is not only designed with many security features to prevent counterfeiting, but also provided different users’ authority management (*i.e.*, read only or read/write to NHI-IC card). Its contents can be read by a card reader. For protecting transaction security, we adopted a secured and encrypted virtual private network (VPN) to connect NHI-IC card readers and data servers. Each card reader has a security authentication module (SAM) and a dual card system. The SAM can perform mutual authentication and secure messaging between the card readers and data servers over the VPN. The dual card system allows a physician to access a patient’s NHI-IC card only when both the patient’s NHI-IC card and the physician’s medical professional cards are present in the card reader. Thus, with these controlled security mechanisms we can assure the confidentiality, integrity, identity verification and non-repudiation of NHI-IC card transactions [32].

According to the Health Insurance Portability and Accountability Act and the Personal Information Protection Act [33,34], minimum necessary medication information was stored, used and disclosed for purposes of treatment to protect patients’ privacy rights in this study. When a patient receives a medical care service, the system will check the health professional card automatically to confirm the health professional card holder and healthcare provider are the same person the patient’s is visiting. If not, the healthcare provider cannot get permission to read or write a medical record. Furthermore, a system operation tracking log was developed in this study. All events of the medical system operation will be monitored and recorded to protect patient privacy.

The prescribed medicines stored in a NHI-IC card are represented by NHI codes. However, a CPOE system adopted by a hospital usually contains drug codes defined by the hospital itself (or hospital codes). Thus, we adopted the Anatomical Therapeutic Chemical (ATC) classification system as a common model for cross-mapping between NHI codes and hospital codes [30]. In the ATC classification system [35], drugs are classified in groups at five levels (from first to fifth levels) based on the organ or system on which they act and their chemical, pharmacological and therapeutic properties. Assignment of drugs to the same ATC code indicates that they are assigned to the same chemical/pharmacological/therapeutic subgroup. Each NHI code can be assigned to a code in the ATC classification system [36].

The NHI-ATC mapping database is used to map a NHI drug code to the corresponding ATC code. The Local-NHI mapping database is used to map a hospital code to the corresponding NHI code. Each drug represented by a hospital code can be mapped to a unique NHI code. Through the Local-NHI and NHI-ATC databases, both hospital codes and NHI codes can be mapped to the corresponding ATC codes. All of the mapping is simple one-to-one mapping. Therefore, DDIs checking is effective and executed without adding complexity. Thus, the prescribed medicines stored in NHI-IC cards can be integrated as a part of the patient’s medication history, which can be accessed by the enhanced CPOE system. Based on the patient’s medication history, the system can detect potential DDIs when a physician prescribes new medications for the patient, no matter which hospital he/she goes to.

## 3. Implementation of Potential DDI Detection across Hospitals

### 3.1. Creation of DDI Databases

The enhanced CPOE system was designed for Taipei Medical University Wan-Fang Hospital (TMUWFH), a teaching affiliate hospital with 750 beds, for outpatient services. The DDI database was created and reviewed by the Pharmacy & Therapeutics Committee of TMUWFH based on the drugs the hospital used, the clinicians’ suggestions and references to the commercial databases and guidelines such as MICROMEDEX^®^ and Drug Interaction Facts [37]. The drug items were coded with their corresponding ATC codes. Each drug pair was associated with a severity level of the interaction, as assigned by Hansten and Horn [38].

(1) Severe interaction (“Avoid administration of the combination.”);
(2) Moderate interaction (“Avoid administration unless it is determined that the benefit of co-administration outweighs the risk to the patient.”);
(3) Mild interaction (“Minimize risk by considering alternative agents or change dosage or route of administration.”).

In addition to the level of severity, each DDI drug pair was also associated with DDI mechanisms, affecting time (onset), warning messages and suggestions to clinicians. To avoid producing too many alerts [39,40,41], the Committee decided to exclude most mild interactions, and agreed on the minimum set of drug pairs that were currently used in the hospital that could lead to potentially significant DDIs. There were 360 drug pairs in the DDI database including 94 severe, 262 moderate and four mild interactions.

### 3.2. Detection of Potential DDIs Involving Multiple Hospitals

To detect potential DDIs, we compare each medicine to be prescribed by the patient’s doctor in this visit with all the medicines that were stored in the patient’s NHI-IC card and that are currently taken by the patient. If a comparison (a drug pair) can be found in the DDI database, the drug pair is potentially interact with each other. A medicine is identified as “currently-taken” if the prescribed date of the medicine plus the prescribed interval of days is equal to or greater than the current visit date. Only those medicines currently-being-taken will be checked against the medicines to be prescribed at this visit.

The algorithm for detection of potential DDIs across hospitals can be described as following. Let PCTDS represent a set of medicines currently being taken that were read from a patient’s NHI-IC card, and TBPDS represent a set of the medicines that are to be prescribed by a doctor at this visit. Let x.u be a drug item x with its attribute (field) u. Let PDDIDS represent a set of potential DDI drug pairs that are detected at this visit, initially PDDIDS is empty.

The drug codes of medicines in PCTDS and TBPDS are represented with NHI-codes and hospital-codes, respectively. They are mapped to the corresponding ATC-codes so that drug-pairs that are stored in PDDIDS are represented by ATC-codes. The algorithm for detection of potential DDIs across hospitals can be described as following.

1. Map all medicines in PCTDS and TBPDS to their corresponding ATC codes.
2. Make drug-pairs (one from PCTDS, the other from TBPDS) and perform DDI checks
   FOR each record *x* ∊ PCTDS, DO
    FOR each record *y* ∊ TBPDS, DO
     IF (*x*.ATC-code, *y*.ATC-code) or (*y*.ATC-code, *x*.ATC-code) can be found in the
      PDDIDS,
     THEN create a record *z* in the PDDIDS, where
      *z*.ATC-code = *x*.ATC-code
      *z*. ATC-code = *y*.ATC-code
     END
    END
   END

The algorithm shown above firstly converts different drug-code representations to a common representation, the ATC coding system. Then it makes drug-pairings using one drug item from the medicines currently being taken that were previously prescribed and stored in a NHI-IC card, and the other from the medicines that are to be prescribed at this visit. Lastly, the algorithm performs DDI checking to see if the drug-pairs can be found in the DDI database. The potential DDI drug-pairs are stored in PDDIDS dataset.

### 3.3. Integration of the Potential DDI Detection Algorithm into Outpatient Workflows

**Figure 2:**
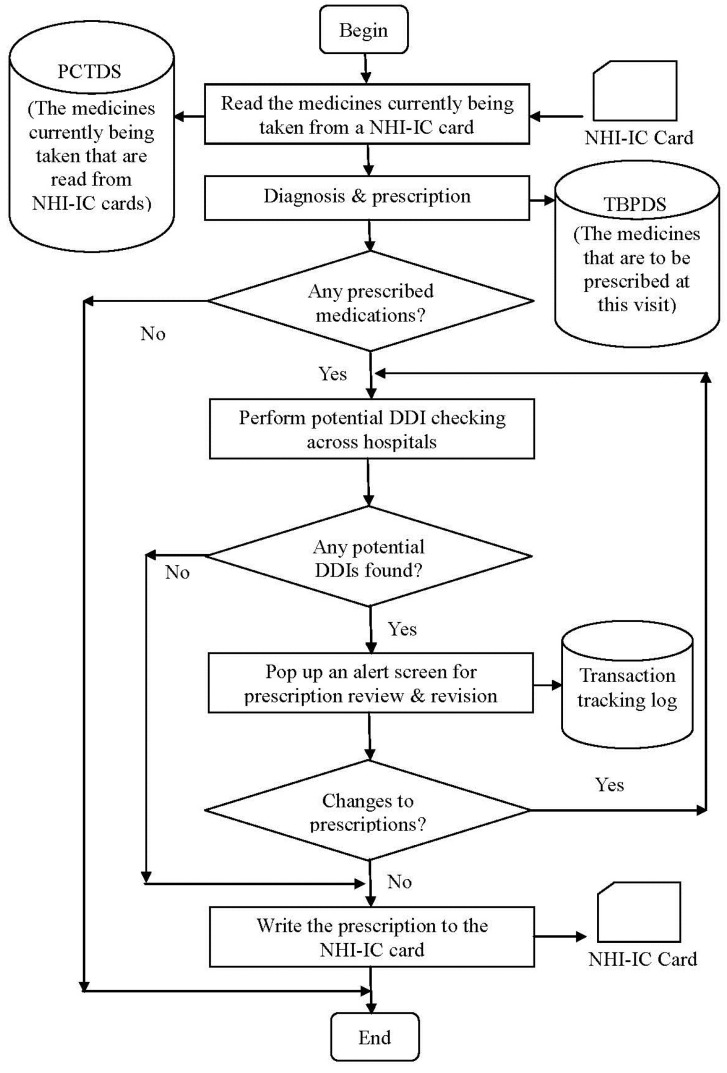
The workflow for detecting potential DDIs involving multiple hospitals.

The workflow of outpatient services to detect potential DDIs for patients who receive medication care from multiple hospitals is shown in Figure 2. For each patient to receive a medical care service, they must present their NHI-IC card to their doctor. The doctor operates the enhanced CPOE system to read the medication history from the patient’s NHI-IC card. If there are medicines currently being taken, they are stored in the PCTDS dataset. After making a diagnosis for the patient, the doctor may prescribe medicines for the patient using the CPOE system. If there are medicines to be prescribed (TBP medicines), they are stored in the TBPDS dataset.

Following this, there are two phases of potential DDI checks. In the first phase, DDI checking within a hospital, we compare each of the TBP medicines with the one that is currently being taken by the patient, and was prescribed at previous visits to this hospital. Since most CPOE systems can handle DDI checking within a hospital [24,42,43], in this study we will not explore this phase further. In the second phase, DDI checking across hospitals, we examine pairs of drugs: one selected from the TBPDS, and the other selected from the PCTDS. Each drug-pair is then checked against the DDI database. The drug-pair is said to be a potential DDI if it is present in the DDI database. The potential DDI drug-pair is then stored in the PDDIDS dataset.

**Figure 3:**
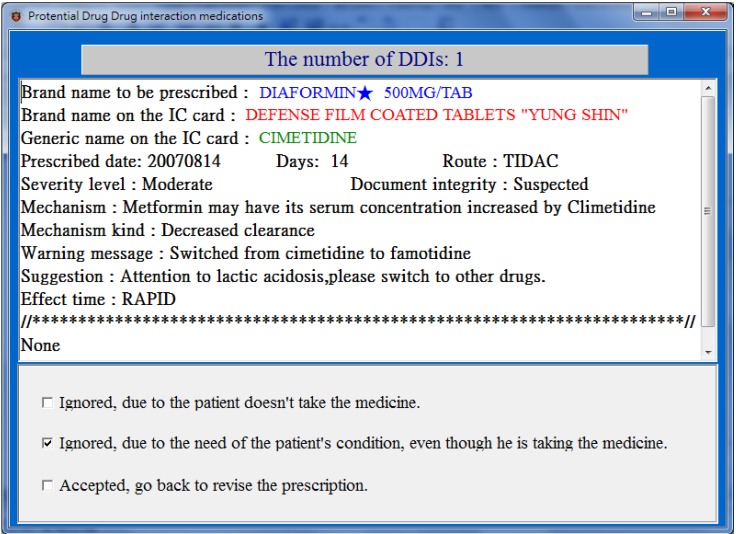
A popup screen (translated from Chinese) showing potential DDIs when prescribing.

For each drug-pair in the PDDIDS dataset, the CPOE system signals the doctor and opens a screen (Figure 3) displaying (1) the brand name of the medicine to be prescribed, (2) information regarding the medicine currently being taken including the generic name, the brand name, the date it was prescribed, the dose and route of administration, and (3) the DDI information including the level of severity, the mechanism of the DDI, effect time, warning message and suggestions. The doctor can then review the prescription with the patient, and take an appropriate action from the following:

(1) Ignored, the patient did not take the medication, or ignored for short;
(2) Ignored, due to the need of patients’ conditions, or ignored but needed for short;
(3) Accepted, go back to revise the prescription, or revised for short.

The actions taken by the doctor and the changes to the prescription are recorded into a “transaction tracking log” for further analysis. Finally, after all the drug pairs have been reviewed and the prescription finalized, the prescribed medicines are sent to Billing Department where cashiers collect all charges and write the prescribed medicines into the patient’s NHI-IC card.

## 4. Investigation of Physicians’ Responses to DDIs Checking across Hospitals: An Empirical Study

To investigate physicians’ responses to DDIs checking across hospitals we focused on three major aspects: (1) physicians’ attitude to access patients’ medication history stored in NHI-IC cards, (2) the capacity adequacy of NHI-IC cards for DDI checking across hospitals, and (3) the physicians’ response to potential DDI alerts. Thus, we developed a transaction tracking log to keep track of physicians’ operations on NHI-IC cards and their actions taken to pop-up screens that showed up when potential DDIs were detected. The transaction tracking log was integrated into the CPOE system, and could be activated/deactivated when needed.

The empirical study was conducted in the Department of Endocrinology & Metabolism (E&M) of the TMUWFH from 1 August to 31 October 2007. This Department was chosen because patients who visit were more likely to have chronic diseases and seek medical care at different hospitals. We activated the transaction tracking log during the period of the study. The log was analyzed after the study using descriptive statistical method. Owing to the importance of protecting the participants’ rights and privacy, a formal ethics-based approval of this study was obtained from the Institutional Review Board (IRB) by the Institutional Review Board at the TMUWFH.

A summary of the log analysis is shown in Table 1. There were 11,612 patient visits to the Department over the three months. In 71.01% (8,246) of patient visits, physicians read the patients’ NHI-IC cards. 33.02% (2723) of the card reads contained data of at least one medicine currently being taken. During the study period, 21684 medicines that were currently being taken were read from the NHI-IC cards in total, 17985 (82.94%) of which were prescribed at the last visit. This implies that if a patient’s medicines prescribed at the last visit are fully recorded in patient’s NHI-IC card, then NHI-IC card contains at least 80% of the currently-taken medicines, no matter how long the prescribed medicines can be stored in the card.

**Table 1:** The analysis summary of NHI-IC card reads.

Month	(a) Patient Visits	(b) NHI-IC Card Reads (% *)	(c) # of Reads with Currently-Taken Medicines (% **)	(d) # of Currently-Taken Medicines	(e) The Currently-Taken Medicines that were Prescribed at the Last Visit (% ***)
Aug.	3,853	2,737 (71.03%)	849 (31.02%)	7,220	6,243 (86.47%)
Sep.	3,632	2,617 (72.05%)	903 (34.51%)	7,038	5,830 (82.84%)
Oct.	4,127	2,892 (70.08%)	971 (31.71%)	7,426	5,912 (79.61%)
Total	11,612	8,246 (71.01%)	2723 (33.02%)	21,684	17,985 (82.94%)

*: (b) ÷ (a) × 100; **: (c) ÷ (a) × 100; ***: (e) ÷ (d) × 100.

The analysis of physicians’ responses to pop-up screens when potential DDIs were detected is shown in Table 2. In total, there were 10,036 prescriptions issued to 11,612 patient visits over the three months. Among the issued prescriptions, there were 7 (0.09%) prescriptions that had at least one drug item that might interact with the medicines currently being taken stored in NHI-IC cards. Each alert prompted a screen to pop up for a physician to review. One alert was ignored (due to not being taken), four were ignored but needed (due to the need of the patient’s condition), and two were accepted to revise the prescriptions. All the alerts were responded to properly, none were closed directly after the alert screen popped up.

**Table 2:** The analysis of alerts and physicians’ responses.

Month	Total Prescriptions	NHI-IC Card Reads	Potential DDIs Alerts (% *)	Actions Taken by Physicians		
				Ignored (not Taken)	Ignored but Needed	Revised
Aug.	3,304	2,737	2 (0.24%)	0 (0.00%)	2 (100.0%)	0 (0.00%)
Sep.	3,169	2,617	3 (0.33%)	1 (33.33%)	1 (33.33%)	1 (33.33%)
Oct.	3,563	2,892	2 (0.21%)	0 (0.00%)	1 (50.00%)	1 (50.00%)
Total	10,036	8,246	7 (0.09%)	1 (14.29%)	4 (57.14%)	2 (28.57%)

* DDI alerts ÷ Total prescriptions × 100.

These alerts signaled physicians about the drug items that might interact with those prescribed by outside hospitals. Of these DDI alerts, six were at moderate level and one at severe level, as shown in Table 3. The most frequently seen DDI pair was metformin HCl and cimetidine, which happened four times. This is reasonable because many patients who visit the Department of Endocrinology & Metabolism are diabetics and have kidney conditions.

**Table 3:** The occurrences of DDI drug pairs during the study period.

Generic Name	ATC Code	Generic Name	ATC Code	Severity Level	Occurrence
METFORMIN HCL	A10BA02	CIMETIDINE	A02BA01	Moderate	4
GLYCYRRHIZA EXTRACT	R05FA02	IMIPRAMINE HCL	N06AA02	Moderate	1
GEMFIBROZIL	C10AB04	WARFARIN	B01AA03	Severe	1
PROPRANOLOL HCL	C07AA05	FLUOXETINE (HCL)	N06AB03	Moderate	1

## 5. Discussion

Drug-drug interactions are of potential concern whenever a person takes two or more medicines. Although comprehensive medication reviews are effective, they are very labor-intensive and costly. Patients might not always receive their medication care from the same pharmacy or hospital. CPOE systems were developed to support prescriptions, however, most are designed for use in a single hospital or one managed care organization setting and cannot perform DDI checks beyond the boundaries of hospitals due to lack of a common infrastructure for sharing a patient’s medication history [20,21,22,23,24,25]. The Taiwanese Ministry of Health and Welfare has established a web-based DDI database system, which allows medical professionals to check medications in a prescription on DDIs manually [44]. The system is stand alone, not a part of CPOE system. For every check, a medical professional must switch his/her work from CPOE to the DDI database system, and input all the medication items prescribed at a prescription. Due to huge volume of outpatients, it is impossible for physicians to use it under such time constraints. Our approach made use of NHI-IC cards and the ATC system as common infrastructures for sharing a patient’s medication history among hospitals in Taiwan. The NHI-IC cards were used to carry the medication history, and the ATC system provided a unified drug classification scheme for mapping prescribed medicines among different hospitals. All of the mapping is simple one-to-one mapping. The response time in a massive ADRs checking condition is about 16 s. Therefore, DDIs checking is effective and executed without adding complexity. Thus, given that the CPOE system was enhanced by the accessibility to NHI-IC cards, this allowed physicians to check for potential DDIs across hospitals when prescribing. Hence, our approach can improve patient safety, particularly for patients who receive medication care from different hospitals.

Based on a study on incidence of clinically relevant potential drug-drug interactions using a drug claims database [45] 0.2% of total prescription claims were potential DDIs that were identified by a sophisticated DDI filtering program, and 0.04% of total prescription claims were clinically relevant DDIs that were identified by the combination of the DDI filtering program and clinical pharmacist review. The enhanced CPOE system presented here produced seven alerts (0.07% of total prescriptions) over 3 months of the study period (Table 2). The rate is higher than that identified by using the combination of the DDI filtering program and clinical pharmacist review, but less than that identified by only using a sophisticated DDI filtering program. This result might also have been affected by the DDI database which contained the minimum set of clinically significant DDI drug-pairs, consequently, fewer alerts were generated.

Even though inclusion of NHI-IC cards into medical services may increase service time [26], this study shows that during most patient visits (71.01%), physicians read patients’ NHI-IC cards. This implies that most physicians would use NHI-IC cards to better protect patients from the risk of DDIs even though it requires a little additional effort. We also make changes to the procedure of writing NHI-IC cards. Initially the card writing procedure was performed by physicians after their prescriptions were completed. After the change, the procedure was performed by cashiers at checkout counters. This change reduced the physicians’ workload and service time. Thus, we suggest minimizing physicians’ administration burden whenever possible when adopting smart cards for storage of patients’ medication history.

As indicated in Table 1, if a patient’s prescribed medicines at the last visit were recorded in NHI-IC cards, the cards could contain 82.94% of medicines currently being taken in his/her medication history. According to the studies [46], in most cases the number of medicines prescribed at an outpatient visit is less than 6. There are 60 entries reserved for the storage of medication history in a NHI-IC card. Thus, the reserved storage space of an NHI-IC card is adequate to accommodate information regarding a patient’s medicines that might be prescribed at different hospitals.

Based on the NHI reimbursement policy, only the medications covered by the insurance program must be written into NHI-IC cards. Self-paid medicines are not recorded on NHI-IC cards. There are very few medications (<5%) which are not covered by the NHI program [29]. Moreover, NHI-IC cards have been commonly in use to access medical services in Taiwan, Thus, it is a good choice to use the NHI-IC cards as a common infrastructure for detecting potential DDIs across hospitals. However, in about 29% of all patient visits, physicians didn’t perform NHI-IC card reads even though we improved the workflow. Further improvement to the workflow is needed to increase the percentage of card reads. For example, in most cases, patients have a waiting period before seeing their doctors, and NHI-IC card reads could be performed during the waiting time. However, such a change involves many stakeholders (patients, physicians, administration staff and nurses) and more work is needed to continue to seek appropriate technologies and redesign the outpatient workflow to minimize medical professionals’ burden and meet the different stakeholders’ expectations.

## 6. Conclusions

Our approach adopted NHI-IC cards as a common medium for carrying patients’ medication history across hospitals in Taiwan. The ATC system was used as a unified drug classification scheme for cross-mapping between NHI codes and hospital codes among different hospitals. All of the mapping is simple one-to-one mapping, therefore, DDI checking is effective and was executed without adding complexity. Given that the CPOE system was enhanced by the accessibility to NHI-IC cards, this, allowed physicians to share patients’ medication history across hospitals. Our approach can help physicians detect potential DDIs across hospitals and provide safer medication care, particularly for the patients who receive medication care from multiple hospitals. Although physicians read patients’ NHI-IC cards at most patient visits (71.01%), there is still room for improvement. We need to continue improving the outpatient workflow and seek appropriate technologies to minimize medical professionals’ burden and to meet different stakeholders’ expectations.
